# Balancing Performing and Teaching Roles: The Voice of Classical Singers

**DOI:** 10.3389/fpsyg.2018.02503

**Published:** 2018-12-13

**Authors:** Christina Raphaëlle Haldane

**Affiliations:** Faculty of Music, University of Toronto, Toronto, ON, Canada

**Keywords:** qualitative research, classical singers, balancing, performing, teaching, voice, vocal pedagogy

## Abstract

How do classical singers combine performing and teaching, two highly challenging and consuming careers? The life of a performer combines reward with intense challenging commitment. Furthermore, for the classical vocalist whose body is the instrument, maintaining good health is a priority. Teachers of singing have demanding roles, with the responsibility of guiding their students' vocal technique, in addition to providing inspiration, emotional support and career guidance. Moreover, their work can be taxing on their voices. There is research pertaining to musicians who balance teaching and performing, however the literature reviewed did not present a study which focused solely on classical singers who also teach, whose operatic engagements can be lengthy and travel-orientated. This gap in the literature provided an opportunity to contribute further to the field and examine the relationship between successfully balancing a performance and teaching career for classical vocalists. My aim was to explore: (1) how classical vocalists with extensive performance schedules maintain a commitment to their teaching studio; and (2) how teachers of singing who manage large private studios, and/or teach at high-level music institutions, balance performance careers with their responsibilities to their students. A phenomenological approach was selected for my exploratory study, using a qualitative method to devise an interview guide and analyze data. The procedure used for selecting the sample group was to invite participants representing a range of professional involvement in both teaching and performing. Diversity with regards to gender, base location and experience was also considered. Participants mainly responded via e-mail interviews. They were invited to discuss the following themes: balancing performing obligations with commitments to students, benefits of performing on pedagogy, and maintaining vocal health. Overall, participants felt that their performance experience was essential to their work in the teaching studio, with performing seen as a source of learning to be transmitted to students. On the other hand, for some, teaching was also seen as a source of learning, enhancing performances. Although demanding, the benefits of maintaining and enjoying both a teaching role and an active performing life were affirmed by participants. Attempting to balance both roles becomes a constant quest.

## Introduction

As a professional classical singer, and teacher, I have experienced the challenge of balancing both my performing and teaching roles since 2006. Reflecting on my own experience, emerging observations pointed to the lives of performing artists as immensely rewarding, requiring intense focus and commitment, yet also presenting daunting challenges. From my stand point, for the classical vocalist whose body is their instrument, maintaining general, emotional and vocal health becomes a priority. On the other hand, teachers of singing have highly fulfilling and demanding roles, with the responsibility of guiding their students' vocal technique, in addition to providing inspiration, emotional support and career guidance. All the while, their work can be highly taxing on their voices. My query into these issues became focused on the following question: how do classical singers successfully combine these two highly challenging and consuming aspects of their professional lives? Being also engaged in studies and research in the field of voice performance, this question became the impetus to my exploring literature in the field, followed by designing a field study to hear the “voice” of singers of classical music who also teach. This article presents an overview of social studies in the area of musician performance and teaching, and of findings from my own exploratory qualitative study.

## Literature Review

As I started investigating literature related to issues in combining the roles of a performing singer and voice teacher of classical music, I became aware of a lack of studies in this field. I then decided to extend my search, and to review studies on combining musical performance and teaching. This larger scope for my query also enabled me to distance myself from my own experience as a performing singer and voice teacher of classical music. As will be seen below, I found studies relating to a number of themes, some of which are echoed in my own research study. For instance, is there a predominant role, or are roles intertwined for musicians who perform and teach? How do musicians with demanding schedules deal with maintaining good health as well as continuing to train in their field?

There is research pertaining to musicians who balance high-level teaching, performance obligations, and other roles within their professional practice. Some studies present how musicians navigate their multiple roles (Miller, 2000; Rice-See, 2003; Chapman, 2006; Triantafyllaki, 2010; Bennett, 2016), while in other studies the concept of musicians' intertwined roles has evolved (Bennett, 2016; Brinck, 2018). There are studies which examine what students expect from their mentors (Mills, 2002; Bennett and Stanberg, 2006). Furthermore, the literature outlines how musicians maintain their health (Teague and Smith, 2015; Bennett, 2016), perceive their identity (Mills and Smith, 2002; Huhtanen, 2004; Teague and Smith, 2015; Bennett, 2016), and adapt to the challenges they face (Smilde, 2011; Burnard and Haddon, 2015; Bennett, 2016; Kresek, 2018). Finally, some of the themes presented relate to the need for training and professional development (Bennett and Stanberg, 2006; Burnard, 2012; Varvarigou et al., 2014; Haddon and Burnard, 2015; Bennett, 2016; Hennekam and Bennett, 2017). The literature review explores findings under these themes and concludes with a perceived gap regarding issues viewed and experienced by classical singers who also assume the role of teachers.

### Predominant Role

A survey examining the balance between performing and teaching was conducted by Lyn Rice-See, amongst pianists holding faculty positions at higher education institutions in the (Rice-See, 2003, p. 30). Although not classical vocalists, pianists share commonalties, especially with regards to the demands associated with memorizing music and the time commitment needed to enjoy a successful performance career, thus making them a viable comparison group with singers.

More than half of respondents (66%) in Rice-See's survey identified themselves as teachers, who felt that performing regularly was essential to their musical life (Rice-See, 2003, p. 31). On the other side of the spectrum, about a fifth of respondents identified themselves first and foremost as performers who also teach, and about a tenth of respondents identified as teachers who only performed occasionally (Rice-See, 2003, p. 31–32). Many respondents were adamant that their performance activities enhanced their teaching, as they were leading by example and sharing their experiences (Rice-See, 2003, p. 31). More than half of the respondents discussed frustration with their schedules, highlighting the dichotomy of giving their all to both teaching and performing, and the challenges posed to finding the time to simultaneously dedicate to both with equal vigor (Rice-See, 2003, p. 32). Some respondents who rarely performed reported making conscious decisions to focus far more on their students, as performance obligations caused their teaching to suffer (Rice-See, 2003, p. 32).

Rice-See's research also shows a correlation between the way respondents viewed their own mentors and how they evaluated themselves as teachers or performers. The largest number of respondents at 65% reported that they thought of their mentors as teachers who performed regularly, with most of that group viewing themselves in the same way (Rice-See, 2003, p. 32).

The findings in Rice-See's research present a variety of attitudes toward combining performance with teaching. Although the survey group all held demanding high-level teaching positions, the majority felt their performance activities were vital to their teaching. Furthermore, more than half of respondents at 54% wished for more time to dedicate to performance, confirming that the link between the stage and the studio is strong (Rice-See, 2003, p. 32).

Musicians have outlined how challenging it is for them to keep motivated and to cultivate the high technical proficiency required for performing, whilst actively engaged in other roles (Covington, 1983; Maehr, 1983, cited by Bennett, 2016, p. 86). Dawn Bennett conducted research with classically trained instrumental musicians and explored the correlation between their perceived notions of success and identity, through the framework of their non-performance and performance activities, which when combined contributed to their professional careers (Bennett, 2016, p. 3, 5). Musicians were selected internationally, and were based in the U.K., Australia, U.S.A., Asia and Europe, with a total of 159 participants in the survey or interviews (Bennett, 2016, p. 102). The findings showed that musicians spent more time teaching than in any other professional activity, with 82% of participants teaching, and 70% performing, as part of their professional roles. Only 8% engaged solely in performing (Bennett, 2016, p. 103–105). Participants highlighted the benefits of teaching, which provided financial stability and control over their schedules and artistry, however 33% noted the need for an improved balance between their teaching and performing roles (Bennett, 2016, p. 108).

Angeliki Triantafyllaki surveyed the concept of identity and professional knowledge with regards to advanced music teachers, in both a Conservatory and a University Music Department in Greece, providing some contrasting findings pertaining to the importance of performance on teaching expertise. Her research shows that the majority of the Conservatory teachers separated their studio success from the stage, claiming the lifestyle of a performer was not conducive to being there for students, and furthermore, that performers did not necessarily have the pedagogical skills to expertly pass on their knowledge (Triantafyllaki, 2010, p. 83).

Richard Miller had an international performance career as a tenor soloist, authored vocal pedagogy books and articles, and taught singing in the studio and masterclasses settings. His work as a singing teacher and his scientific approach to vocal pedagogy have made him an authoritative figure on the subject. He advocates that effective instruction relies on the teachers' ability to decipher subtle perceptions of tone and their ability to guide their students to be aware of those subtleties themselves (Miller, 2000, p. 42). Furthermore, Miller proposes that “well-informed pedagogues will not impose their own sensations and experiences on the student seeking specific and artistic advice” (Miller, 2000, p. 43). Miller argues that for best practice, teachers of singing should base their instruction on an understanding of the scientific facts behind how phonation and resonance are produced in the body, and not rely on vague concepts such as imagery, and their own interpretations of what they physically experience when they sing (Miller, 2000, p. 43). A performance career may contribute to the qualities Miller attributes to a successful teacher. However, performing may not be essential to his description of a skilled voice pedagogue, which relies on a scientific approach and an ear which can perceive the subtleties of the ideal vocal tones appropriate for a classically trained singer.

In addition to positive performance experiences, negative experiences can also contribute to success in the studio. Janice Chapman discovered her skill and curiosity about teaching, upon the realization that her own vocal technique was faulty and causing her difficulties in her performance career (Chapman, 2006, p. 5). In her book *Singing and Teaching* Chapman freely discusses how learning to help herself through vocal problems led to her own expertise as a teacher, thus turning a negative into a positive (Chapman, 2006, p. 5).

Following on the studies above which focused on identifying a predominant role for musicians and classical singers who also teach, the studies presented in the next section explore more in depth the dual roles adopted by these professionals and point to these roles being intertwined.

### Intertwined Roles

In higher music education there can be a dichotomy with regards to what constitutes the ideal applied studio teacher, with respect to their pedagogical and performance background. Many higher music institutions seek elite performers to fulfill teaching positions, however expect them to also successfully navigate academic and administrative roles (Bennett, 2016, p. 143). Furthermore, some conservatories employ elite performers as teachers, regardless of pedagogical experience or credentials (Bennett, 2016, p. 63). On the other hand, there can be negative perceptions about those who predominantly teach, regarding it as a “fall-back career” for those whose performance aspirations were not fulfilled (Bennett, 2016, p. 87). To counter the notion of dichotomy, Gareth Dylan Smith argues that the separation of fine pedagogy and musical excellence is futile, as they are one and the same: skill sets are combined as musicians *are* teachers, and vice-versa (Smith, 2018, p. 40). Lars Brinck highlights how forms of musical participation, led by teachers who frame their pedagogical approach through performance-led activities, can provide their students with “access” to enhanced musical knowledge (Brinck, 2018, p. 200). The “musician-teacher” roles are intertwined; teachers who are musicians have pedagogical tools at their disposal, and musicians who teach bring their performance experience and artistry into their classrooms and studios (Brinck, 2018, p. 200).

As will be seen below in the current qualitative study with classical singers who also teach, identifying a predominant role or merging both roles are also commented on by participants.

Other studies, presented in the next section, explore the views of music students toward their teachers as another way of clarifying the dual roles of performer and teacher. However, this theme was not explored in the current qualitative study with classical singers who also teach.

### What do Students Want?

Janet Mills of the Royal College of Music, London, an internationally recognized performance conservatory for musicians, conducted research on students' perceptions of successful instrumental and vocal tuition at the conservatory-level in the UK. Relating to the performance level of instructors, her findings show that students felt they benefited by being taught by instructors who were currently, or in the past, at the top of their profession as performers (Mills, 2002, p. 79). Additionally, students reported that they wanted their lessons to be regular, and for teachers to be involved in both musical and personal development (Mills, 2002, p. 79). Students value both performance expertise and continuity with regards to lesson scheduling and support. This poses a challenge for the teacher who performs, to provide both regular instruction and mentorship, whilst negotiating the travel and time needed for an elite performing career.

Furthermore, some music students have expressed worries associated with taking on potential teaching roles, as they feel their performing standards could diminish as a result, and that teaching hours could conflict with performance opportunities (Bennett and Stanberg, 2006, p. 222).

In the next two sections, the notion of health is explored as well as adaptation mechanisms used by musicians to preserve their identity when faced with the demands of combining performance and teaching.

### Health, Identity, and Adaptation

Maintaining physical and mental health also poses a challenge for musicians engaged in multiple roles, including performing and teaching (Teague and Smith, 2015, p. 181, 182). The challenges musicians face whilst balancing their performing obligations such as touring, irregular hours and travel, teaching commitments and family life, can lead them to be susceptible to decreased mental and physical health (Teague and Smith, 2015, p. 181, 182). Musicians are more prone to injuries than athletes, and their mental and physical health can be vulnerable, especially during periods of intense engagement such as extended orchestral commitments, and competitions (Bennett, 2016, p. 49).

Given the variety of roles that musicians engage in, issues related to the concept of *identity* are also explored in the literature. Dawn Bennett's research illustrates that for musicians who are active in multiple roles beyond that of their applied performance practice, self-identity becomes more intricate than simply identifying with one role or another (Bennett, 2016, p. 8). She advocates that career fulfillment is closely linked to one's own psychological lens on personal success, as experienced in the various roles undertaken within the umbrella of being a “musician” (Bennett, 2016, p. 2). Through personal experience and her research on pianists who transitioned into teaching, Kaija Huhtanen concluded that the loss of performance orientated ambitions could lead to feelings of failure. She separated her participants into two groups: those who were content to identify as teachers within their professional roles, and those who viewed teaching as merely an income stream whilst holding onto their performance goals, and who thus developed difficult associations with their playing as a result (Huhtanen, 2004, cited by Bennett and Stanberg, 2006, p. 220). On the other hand, Janet Mills and Jan Smith's research on Conservatory graduates concludes that many musicians do not identify with the role or roles, that provide their income, thereby linking their identity to their aspirations over their objective activities (Mills and Smith, 2002, cited by Bennett, 2016, p. 88). Family life was firmly situated within the frame of identity by the musicians in Adele Teague and Gareth Dylan Smith's research on portfolio careers and work-life balance. Participants within their study who had children highlighted how their primary identity lay with their families, however the one participant without children identified more strongly with the role of musician, whilst still considering any potential family obligations as a strong factor in future career choices (Teague and Smith, 2015, p. 185).

The ways in which musicians must adapt to various roles within their practice is a theme presented in the literature. Dawn Bennett describes musicians as having to create “protean” or “portfolio” careers, in which they constantly evolve their practice and simultaneously undertake different roles (Bennett, 2016, p. 9). Katherine Kresek characterizes freelance musicians as operating within “nomadic conditions” as they balance their careers as both performers and teachers (Kresek, 2018, p. 171). Musicians must accommodate their obligations to various organizations, be it through their performance work or itinerant teaching commitments, whilst negotiating their own attitudes on performance and pedagogy with those of their professional partnerships (Kresek, 2018, p. 171). Pamela Burnard and Elizabeth Haddon suggest that for musicians, professional success is defined by the way they employ various “musical creativities” within their practice (Burnard and Haddon, 2015, p. 4). In order for musicians to navigate the complexities of their various professional roles, they must be adaptable, contemplative, creative, successfully work in partnerships and develop entrepreneurial and business acumen (Smilde, 2011, cited by Helfter and Ilari, 2015, p. 137).

As can be seen above, enhancement of health and adaptive mechanisms can support musicians' identity. As will be explained below, the concept of identity was not explored in detail in the current qualitative study I conducted. However, health and adaptive mechanisms were commented on by participants. In the next section, studies related to the positive effects of training and professional development are explored and linked to an enlargement of possible roles.

### Training and Professional Development

The positive influence of pedagogical experience is suggested by Janet Ritterman. She recommends that pedagogical training can help music students to become more proficient learners through their understanding of teaching practice (Mark, 1998, cited by Bennett, 2016, p. 74). Enhanced learning skills can in turn lead to the further development of performance practice.

Exposure to teaching has led to a positive evolution of perceptions with regards to the career aspirations of performance-oriented music students. Maria Varvarigou et al.'s research on the “LSO On Track” partnership between the Guildhall School of Music, the London Symphony Orchestra and East London Music Services revealed some important findings. On Track enabled the Conservatory students and professional musicians from the orchestra to work with school-aged students from London's East End in a series of workshops, school performances and rehearsals/sectionals (Varvarigou et al., 2014, p. 85). Many of the Guildhall School of Music participants highlighted how their experience on the scheme had fueled a newly found interest in teaching, and that they were actively considering teaching roles as a part of their performance-oriented career goals (Varvarigou et al., 2014, p. 89). Dawn Bennett and Andrea Stanberg surveyed the perceptions that students of education, performance and composition from the School of Music at the University of Western Australia experienced with regards to teaching roles prior to, and after they attended a 12-week pedagogy seminar as part of their undergraduate degree (Bennett and Stanberg, 2006, p. 221). Prior to the seminar, 53% of the participants who were performance majors reported that a motivation for teaching was linked with a potential lack of performance opportunities (Bennett and Stanberg, 2006, p. 223). However, after completion of the pedagogy seminar, every performance and composition participant reported that their motivations for teaching had evolved and were aligned with the positive experience they associated with the practice, in combination with financial stability as reported by 75% of the participants (Bennett and Stanberg, 2006, p. 223). Positive engagement and perceptions contribute greatly to career aspirations and motivations, with an active “passion for the field” noted as one of the key personal traits attributed to maintaining longevity in a musical career (Bennett, 2016, p. 96).

Evolving entrepreneurial training initiatives for music students engaged in higher education has been advocated by Haddon and Burnard (2015, p. 262, 263) as an essential element for preparing musicians to work professionally in the field. Pamela Burnard suggests that musicians' intrinsic satisfaction can be linked with their development of a “multiplicity of creativities,” which can be a component of successful entrepreneurial practice, possibly outweighing desires for financial rewards (Burnard, 2012, cited by Haddon and Burnard, 2015, p. 270). Dawn Bennett's research highlighted how musicians felt that a key aspect of their long-term career management was acquiring the business and entrepreneurial acumen for realizing their creative goals, successfully marketing and communicating their product, and learning the legal, policy, and tax frameworks in which they operated (Bennett, 2016, p. 93).

The need for continued learning and professional development is another theme presented in the literature. Dawn Bennett's research into musicians' careers highlighted that formal or informal education was essential to managing a long-term career, in addition to maintaining current knowledge of digital technologies and business practices (Bennett, 2016, p. 94). These findings also correlated with Sophie Hennekam and Dawn Bennett's research into those who work in the creative industries. Their research outlined how the need to constantly renew skills posed a challenge for creative industry workers (Hennekam and Bennett, 2017, p. 79). Ongoing training was a key aspect of long-term career management, however it proved costly, and many found it difficult to predict which skill set to focus on, as a result of shifting industry trends (Hennekam and Bennett, 2017, p. 80).

As will be seen below in the current qualitative study with classical singers who also teach, continued learning becomes important in maintaining balance between their two roles.

The literature reviewed as presented above provides insights into how musicians navigate their teaching and performing, and other roles within their practice as well as some of the challenges met in their professional and personal engagement. Additionally, the research reviewed explored concepts of musicians' identity, within both a professional and personal context. Insights on identity within the professional field as well as relating to family life, provided an overview on the complexity of musicians' lives. However, I was foremost interested in investigating how musicians, and specifically classical singers, managed their teaching, and performance roles on a practical level. The literature reviewed did not present a study which focused solely on classical singers, whose operatic engagements can be lengthy and travel-oriented, which can further complicate the issue of negotiating performing with teaching. This gap in the literature provides an opportunity to further contribute to the field and examine the relationship between successfully balancing a performance and teaching career for classical vocalists. Most of the themes discussed in the literature reviewed provide a backdrop to the practical management of roles which I wanted to investigate and document for singers of classical music. Several of the themes reviewed in the literature will be further explored below in the analysis on the findings from the current qualitative study.

## Methodology

### Research Questions

In view of the perceived gap in the literature, discussed above, the following questions were formulated to inform my field research study:

- How do classical vocalists with extensive performance schedules maintain a commitment to their teaching studio?
- How do teachers of singing who manage large private studios, and/or teach at high-level music institutions, balance performance careers with their responsibilities to their students?

### General Approach and Ethics Requirements

A phenomenological approach was selected for my exploratory study into the way classical vocalists balance both their teaching commitments and performance schedules. My objective was to investigate how participants experience combining their active roles as both teachers and performers of singing, in other words, how they think, feel and perceive their experiences (Willig, 2008, p. 69). A qualitative method was used to devise an interview guide, where participants' comments would provide insights into their world. The qualitative method of allowing the participant to express themselves freely under a theme, combined with the role of the author in selecting illustrative quotes, thus highlighting these as the “star attraction” of the study (Chenail et al., 2011, p. 271), supported the aims of my research project. This field study's protocol was approved by the Social Sciences, Humanities and Education Research Ethics Board of the University of Toronto (Reference # 34798). All subjects gave written informed consent in accordance with the Declaration of Helsinki 2013.

The research was conducted as an independent study as part of the requirements for my Doctorate of Musical Arts degree in voice performance at the University of Toronto's Faculty of Music. I have an international career as a classical soloist, in addition to actively balancing my academic life with that of teaching masterclasses and private studio lessons.

### Selecting Participants, Data Collection, Analysis, and Codes Used in Findings

The procedure used in selecting the sample group was to invite participants representing a range of professional involvement in both teaching and performing, focusing on the following categories:

- Predominantly international-level performance career, consistently working at “A” opera houses (“A” opera);
- Predominantly national with occasional international-level performance career (national);
- Predominantly teaching at an institution (institution);
- Predominantly teaching at a private studio (studio);
- National-level, leading roles, musical theater performer and teacher (music theater).

In addition, diversity with regards to gender, base location and experience were also taken into account. Due to my personal experience as a performing singer and voice teacher of classical music, and the time frame of the study, participants were selected from my extended contacts. I had originally planned to interview participants via telephone/Skype or face-to-face. Fifteen potential participants were contacted via e-mail. However, given scheduling, geographical and budgetary restraints, in addition to the time frame of the study, only two participants were interviewed, one via telephone and one face-to-face. For the other participants, eight responded via e-mail interviews.

The e-mail interview process allowed the majority of the participants time to reflect on and express their experiences in their own words, and also to be in control of both the pacing and time frame of their responses (Meho, 2006, p. 1291; Ratislavová and Ratislav, 2014, p. 454). Five participants were based internationally, and two were located in cities that were geographically far from my base within Canada. Given the geographical issues presented, budgetary restraints and short time frame in which to conduct my research, utilizing the e-mail interview allowed me access to a rich sampling of active professional classical singers, which has been highlighted as one of the benefits of this method (Meho, 2006, p. 1293; Burns, 2010, p. 9; Ratislavová and Ratislav, 2014, p. 453, 454). Similar to Edgar Burns' research on utilizing e-mail interviews, the choice of this method evolved through interaction with participants, all of whom were competent and familiar with e-mail and written communications through their administrative work, with some of them highlighting a preference for e-mail over telephone/face-to-face or Skype (Burns, 2010, p. 2). As participants came from my personal extended contacts, trust and the relationship between researcher and participant were established prior to the e-mail interview process, enabling a free flow of information despite the digital medium (Ratislavová and Ratislav, 2014, p. 456). Ethical concerns with regards to managing any potential emotional distress associated with recounting experiences during an interview, which could go undetected without the visual cues of face-to-face interaction, were not a concern given the perceived low risk nature of the subject matter (Ratislavová and Ratislav, 2014, p. 457). Given my engagement in the arena of voice performance and teaching of classical music, e-mail interviewing also established a distance between myself as investigator, and the participants. My involvement was to set open-ended questions as objectively as possible, and leave participants to recount their experience and views in their own space.

Due to the limited number of participants, coding and analysis of the e-mail “transcripts” under different themes were done through a word processing software, without the use of a compiling software such as NVIVO. Tentative themes/codes were used at the beginning of the coding and analysis, and with back and forth refinements these became crystalized as presented in this article. Participants' comments under each theme were collated and counted in order to provide approximate occurrences under the different themes.

In order to provide anonymity and protect personal data, in the compiling of background data and comments, each participant was assigned a pseudonym from Mozart's operas. In this paper, in reporting participants' comments, the pseudonym is used, along with one of the codes listed above regarding predominant professional involvement.

In reporting comments by participants, apart from “all” and “one,” the following expressions are used: comments by 2–3 participants are described as by “a few,” by 4–6 as by “about half,” and by 8–9 as by “almost all.” The expressions “a few” and “almost all” are used similarly in describing aspects of the participants' background.

In quotations, square brackets, ([]), indicate clarifications of the quotation's meaning. Three dots (…) indicate where a sentence or part of it has been removed for the sake of conciseness. Oblique bars (//) indicate where two or three sentences have been removed or where the quotation is taken from two paragraphs in the transcript.

## Findings

### Participants' Background

A range of professional experience was found in the sample group, with singers working predominantly in “A” opera houses, teaching private studios, and performing in music theater (see Table 1 in Appendix 2, containing all tables). Participants were based internationally, in the UK, the Netherlands, Hong Kong, and with 50% from Canada (see Table 2 in Appendix 2). Most of the participants had long careers as performing singers with at least 16 years of experience. A good majority of participants (at 70%) had at least 6 years of teaching experience with a few beginning to teach later in their career (see Table 3 in Appendix 2).

Almost all of the participants trained at postgraduate-level (see Table 4 in Appendix 2). With regards to private teaching vs. teaching at an institution, 50% of participants were affiliated with an institution (see Table 5 in Appendix 2). The amount of time per week devoted to teaching was varied among participants (see Table 6 in Appendix 2), and 70% of them were paid hourly fees (see Table 7 in Appendix 2). The sample group mainly taught at a high-level, with 80% working predominantly with university/conservatory students (see Table 8 in Appendix 2). A good majority of participants (70%) continue to train themselves with lessons and coaching (see Table 9 in Appendix 2).

### Views on Performing and Teaching

Participants were invited to discuss the following themes: balancing performing obligations with commitments to students, benefits of performing on pedagogy, and maintaining vocal health. They were also invited to add additional comments if they wished to do so (see Appendix 1 for interview guide). To contextualize some of the comments presented below, Table 10 in Appendix 2, Appendix 2 provides a summary of each participant's background.

#### Balancing Performing Obligations With Teaching to Students

About half of the participants highlighted that careful planning of performing, practicing, and teaching commitments was essential in establishing the right balance, as detailed below:

*My students work with me because I am a performer. If I need to be away for an audition or performance, I reschedule lessons, or I bring in a pianist/voice coach to take over the lesson while I'm away. I organize my day so that I have the first part of the day for my own practice and preparation, and then teach in the afternoon/evening*. (Countess-studio)

*It can be very tricky fitting everything in, so forward planning and getting lessons in the bank is vital. For instance in the early part of the post-Christmas term, I was teaching 2 days a week to make sure no one fell behind, I am currently away for some time and have put a Dep [a replacement] in for me for 6 lesson periods*. (Marcellina-national)

With regards to their focus on either teaching at an institution or performing in “A” opera houses, participants on these extreme ends of the spectrum, tended to keep their priorities close to their predominant work. Both singers holding full-time professorships at institutions (as detailed in Table 10 in Appendix 2) highlighted their priority to their students:

*[How do I balance my dual roles?] With great difficulty. Students tend to always come first*. (Count-institution)

*When I was teaching, performing obligations were part of my university job. I had to demonstrate my ability to keep a certain level of performance in order to have access to promotions. My commitment to my students always came first*. (Fiordiligi-institution)

Two of the singers with extensive performance careers in “A” opera houses gave their priority to performing obligations:

*Because of my performing life, I don't have time to commit to students: when I'm not working on my singing and I have time … I arrange times to work with the students at [name of institution] when I am free. It's a win-win situation for me at the moment*. (Bartolo-“A” opera)

*I only arrange teaching between [singing] jobs*. (Anna-“A” opera)

As detailed in Table 10 in Appendix 2, these last two participants focus on intermittent teaching which can also be intensive.

One participant brought attention to the difficulties of combining both a busy performing and teaching schedule which included teaching to a wide spectrum of students from adolescents, university/conservatory students to young professionals and adults, as detailed in Table 10 in Appendix 2:

*Can you help me? I can't balance it! It's a learning curve // I am able to, but it takes time*. (Susanna-national)

#### Benefits of Performing on Pedagogy

Although one participant was not currently engaged in performing, all of them emphasized that their role as performers benefited their teaching in the studio, as detailed below:

*I don't think I could teach at all if I hadn't achieved a high level of vocal technique, and if I hadn't been experienced in the performing business. // Every second of my teaching is informed by my performing*. (Cherubino-music theater)

*It definitely benefits, there are a lot of new trends, you keep nourishing yourself, meeting conductors, meeting style specialists, that have new ideas and I think it is extremely important. As a performer you discover everything every time you perform and it triggers ideas for students as well*. (Susanna-national)

*Without performing experience, there is no teaching*. (Zerlina-studio)

*It's total, everything I have learned from my performing is passed on as much as I can to the students whom I am working with. What I am teaching them is what I have learned over the past 35 years*. (Bartolo-“A” opera)

*My performing feeds my teaching … I am constantly working on my technique and continue to train with the top coaches in the US and bring this knowledge and experience to my studio. Because I still take the stage and audition, I can understand the mental work the students need to do on top of their music practice to prepare for their auditions and performances. Because I continue to work on my performance career, I continue to be a very driven and disciplined individual and am a good example of what needs to be prioritized in order to be a classical singer*. (Countess-studio)

#### Maintaining Vocal Health

About half of the participants advocated a vocal warm-up prior to a teaching day, as demonstrated below:

*I find that I have to arrive early enough to fit in some vocal warm-up time prior to my teaching, as constant speaking and demonstrating is very tiring. On days when I don't warm-up I find myself becoming very hoarse*. (Marcellina-national)

*I still warm-up almost every day and try to not speak loudly while teaching*. (Giovanni–“A” opera)

Furthermore, about half of the participants highlighted the importance of maintaining general good health with sufficient sleep and emotional stability, as shown below:

*Technique, priorities and emotional balance*. (Zerlina-studio)

*I need to assure I'm healthy by maintaining a good diet, be sure to have lots of sleep, walk a lot to feel energized and stay calm, avoiding stressful events if possible*. (Fiordiligi-institution)

*Sleep, lots of fluids, and a good dose of NORMAL LIFE!* (Anna-“A” opera)

A few participants also highlighted self-preservation, for example teaching less during busy performance periods:

*This summer … I'll be singing A LOT: 6 days a week. So, then, it'll be about vocal health and conservation more than anything*. (Cherubino-music theater).

Furthermore, efficient planning was advocated by a few participants, with one also suggesting the importance of mental practice, as shown below:

*I have always done a lot of mental practice and make sure that I don't over tax my voice on days when I have to teach. // If I have a concert, I schedule my week as such that I don't take students that week, so that I may keep my voice fresh and my body and mind focused on the performance*. (Countess-studio)

#### Additional Comments

Although participants were not asked about their motivation in teaching apart from their performing role, almost half of them provided comments to that effect.

A few participants accentuated that they felt their teaching life had positive effects on their performing, as indicated below:

*I've always thought that teaching benefits my singing*. (Fiordiligi-institution)

*Teaching others to sing has taught me so much about my own technique. I believe every singer should teach, as we don't even realize how much we do know. It can give us confidence in ourselves as performers*. (Countess-studio)

One participant explained that he enjoyed the status associated with being a professor of singing, in addition to the networking created by bringing his singing contacts to the institution for masterclasses. He also stressed that his teaching life, unlike his performing life, was not for monetary gain:

*I'm not your typical singing teacher. // With my contacts in the business, I can bring well-known singers to come and do masterclasses at the university. I like teaching people who are adult and who I can talk to as an adult and who are quick to understand what I am saying to them. I get paid a bit, I don't make proper money from it … but it's good on the CV!* (Bartolo-“A” opera)

Another participant highlighted his enjoyment of supporting new singers through his teaching role:

*As a teacher, we need to inspire, support, but be realistic when dealing with the different levels of students. Give them opportunities to grow, improve, and the ones that can take advantage of it will be the ones that we can more fully help*. (Giovanni-“A” opera)

Lastly, a few participants emphasized the playful insanity of life as a teaching performer, beautifully surmised in this poem:

*When teaching all hours of the day*,

*My work is like playing, I say*.

While it is a choice

To tend to my voice

*Too often I give it away!* (Count-institution)

## Discussion

### Links to Literature

Differences and commonalties can be established between the current study and the surveyed literature. Not all the pianists in Rice-See's study felt that performing was essential to their teaching, whereas all the singers in the current sample group strongly felt that their performing benefited their teaching. Furthermore, almost all of the singers in the current sample group ensured they were able to be actively performing regardless of their teaching obligations. Commonalties were discovered, as pianists and singers in both studies expressed difficulty in prioritizing both teaching and performance.

Most of the musicians in Dawn Bennett's study incorporated teaching in their professional role, with slightly more than half also performing, however very few were engaged only in performance careers. However, all the singers in the current sample group taught, and almost all were combining their teaching with performing activities, although none were solely engaged in performing. Similarly, to Bennett's study, the singers also highlighted the challenges of balancing both teaching and performing roles within their practice. The musicians in Bennett's study also described the scheduling benefits that teaching allowed them, which correlated with the theme of careful planning outlined by about half of the participants in the current study as well as the two singers who highlighted the flexibility of scheduling students between singing contracts.

Contradictions arose from Angeliki Triantafyllaki's research, featuring teachers at a Greek Conservatory, and findings in the current study on classical singers who also teach. Triantafyllaki's survey emphasized how the majority of teachers at the Conservatory felt the lifestyle of a performer benefited neither students nor a pedagogical approach, whereas all the participants in the current study found their performing to be vital to their teaching style.

Gareth Dylan Smith and Lars Brinck's comments on how both teaching and performing roles are intertwined aligns very well with the findings from the current study, as all of the participants advocated that their performance experience was essential to their teaching. Furthermore, how Janet Ritterman situated pedagogical knowledge as a conduit for enhanced learning also shows similarities to findings in the current study, as a few singers expressed how their teaching positively affected their performing. Additionally, similar to Janice Chapman's study, a few participants in the current study felt that examining their own vocal technique through teaching enabled them to become better singers themselves!

Conservatory students' aspiration to study with teachers who have achieved high-performance levels, exemplified by Janet Mills' research, correlates well with findings in the current study in that all of the participants felt their performing benefited their students. Mills' study also highlights how conservatory students appreciate organized lesson planning and continuity, which emphasizes the theme of careful planning, as reported by participants in the current study. It is valuable to note that the agendas of teachers and students in both studies align well on these two topics.

Exposure to teaching during higher education was shown to affect positively students' perceptions of incorporating the role into their professional practice, explored by Maria Varvarigou et al., Dawn Bennett, and Andrea Stanberg. These findings aligned somewhat with that of the singers in the current study, as they highlighted how they enjoyed their teaching; with a few commenting on how teaching positively affected their performing, and one explaining how he enjoyed the status associated with his professor role and another describing his enjoyment of mentoring young singers. Lastly, a few participants advocated that balancing their performing and teaching roles gave them positive feelings of playfulness and excitement.

Adele Teague, Gareth Dylan Smith, and Dawn Bennett all highlighted the challenges musicians face in keeping physically and mentally healthy, in the contexts of both their professional and personal lives. Maintaining vocal health was a theme that emerged from the current study, with about half of the singers who also teach advocating for vocal warm-ups on teaching days, and routines such as ensuring sufficient sleep patterns and cultivating emotional stability as well as a few highlighting the need for self-preservation during intensive performance commitments.

The need for musicians to develop entrepreneurial skills was discussed in the literature by Elizabeth Haddon, Pamela Burnard, and Dawn Bennett. By managing their active performing and teaching roles on a practical level, whilst continually navigating and establishing successful careers as professional musicians, all of the participants in the current study showed elements of their entrepreneurial acumen. Burnard commented on how satisfaction that is experienced through creative development, which can be situated as an element of entrepreneurial practice, can possibly outweigh desires for financial rewards. This possibility aligns with a comment made by one of the participants in the current study, who highlighted that he did not engage in teaching for monetary gain.

Finally, continued development was a theme that arose from both the literature and the findings in the current study. Sophie Hennekam and Dawn Bennett both commented on how maintaining ongoing training was an essential component of managing a long-term career. Nine of the participants in the current study had postgraduate music degrees, with seven continuing their training with regular singing lessons as well as three engaging in musical development through regular sessions with vocal coaches and conductors. Only two singers in the current study had not engaged in musical training over the past few years (see Tables 4, 9 in Appendix 2).

### Methodological Considerations

The current exploratory study being based on a small sample has limitations in terms of extrapolation to a larger population of classical singers involved in similar roles. However, most of the participants had long performing careers with a good majority of them having also been involved in teaching for several years. Their views were expressed within this context and can help understand the challenges and benefits in combining performance and teaching roles for classical singers.

The data collection method for this qualitative study, using e-mail without a back and forth dialogue can present limitations as more in-depth data could be missed. However, as mentioned above in section Selecting participants, data collection, analysis, and codes used in findings, given the background level experience of participants, and their limited availability, the possibility for them to control medium and message can be seen as a mitigating factor in favor of the chosen procedure. As mentioned above in section Methodology, since I am also involved in the field as a classical singer and teacher, possible influence on my part on their reflective process was also kept at bay and augmented the degree of researcher distance in this stage of the study. Comments by participants were clear enough so that additional interaction with them was not considered necessary.

## Conclusion

All of the participants in the current study felt that their performance experience was essential to their work in the teaching studio, and furthermore the benefits of maintaining and enjoying an active performing life were affirmed by almost all these singers who also teach. Additionally, a few singers highlighted that they felt their teaching benefited their performing and singing technique. There were commonalities shown by participants with very busy performance schedules at “A” opera houses, who accommodated teaching in their scheduling gaps. At the other end of the spectrum, those singers holding positions as full-time professors at institutions and needing to balance their teaching commitments, tended to prioritize their students over their performing. Interestingly, participants on the high pay-scale associated with performing at “A” level opera houses, still chose to teach despite the fact that their monetary needs are met with performing. However, all three of the participants in this category chose to start teaching later in their careers (see Table 10 in Appendix 2).

The findings in the current exploratory study could be the basis for further research. Further studies could include examining in more detail the motivations for teaching, when balancing a studio and performance career poses so many challenges. Studies could also explore whether classical singers who also teach engage in continued training outside the field of purely musical/vocal development as well as how and if they maintain their formal teacher training. Studies could investigate how classical singers who also teach incorporate entrepreneurial practice into their professional careers, and whether they feel they need more guidance in this field. Furthermore, aspects discussed in the literature and not covered in the current study could be explored such as the concept of identity within the umbrella of both their professional and personal lives, examining how performing singers transition into teaching roles, and whether they experience any negative feelings associated with that evolution.

Based on the literature review, findings and discussions for the current study, policy developments could benefit classical singers who also teach. Implications for the future could be to establish for classical singers who also teach more opportunities and support networks and education outside the field of their vocal training, to enable continued development throughout their careers. Subjects for education and support networks could be entrepreneurial development and business skills, navigating physical and mental health as well as opportunities to continually explore teaching experiences and strategies. Furthermore, there is a need to continually enhance funding strategies for professional development, from both governmental and private sectors.

Overall, participants in the current study demonstrated how combining performance and teaching results in joy, creativity, sharing, tensions and adrenaline: all experiences that we as singing artists are drawn to and thrive in.

*It's a crazy life but it's a good one, and it's worth it!!! The performing part is worth it and also the sharing of what you learn along the way is so, so gratifying I find*. (Susanna-national)

## Author Contributions

The author confirms being the sole contributor of this work and has approved it for publication.

### Conflict of Interest Statement

The author declares that the research was conducted in the absence of any commercial or financial relationships that could be construed as a potential conflict of interest.
