# Supplementary material for: Balancing Performing and Teaching Roles: The Voice of Classical Singers
Source: Front Psychol. 2018 Dec 13;9:2503. doi: 10.3389/fpsyg.2018.02503 (PMC6300508; doi:10.3389/fpsyg.2018.02503)
Supplement: Supplementary file 1 [file Data_Sheet_1.pdf]

Appendix 1

Interview guide

1. How long have you been singing professionally?

2. How long have you been teaching professionally?

3. What, if any, is your formal training?

4. What is the ratio of private teaching vs teaching at an institution?

5. How many hours on average do you teach per week? Are you paid per hour, or a salary?

6. What levels do you teach mainly?

7. How do you balance your performing obligations with your commitment to your students?

8. How does your performing experience benefit your teaching?

9. How do you maintain your vocal health when teaching and performing?

10. Do you still take voice lessons yourself?

11. Any further comments for discussion?

Appendix 2

Tables – Participants' background

Table 1: Predominant work focus

Work focus	Number of participants
Singing - international 'A' opera houses	3
Singing - national & some international	2
Teaching - institution	2
Teaching - private studio	2
Music theatre - national	1

Table 2: Base location

Location	Number of participants
Canada	5
Hong Kong	1
Netherlands	1
United Kingdom	3

Table 3: Experience in singing and teaching

Years of experience	Professional singing	Professional teaching
1-5		3
6-10	1	3
11-15		1
16-20	2	
21-25	2	

26-30	1	2
31- 34	2	1
35-40	2	
**Mean**	26 years	14 years

Table 4: Formal training

**Type of training**	**Number of participants**
Postgraduate (M.Mus and above)	9
Private training	1

Table 5: Private versus institutionalized teaching

**Type of teaching**	**Number of participants**
Mostly Institution	5
Mostly Private	2
Almost equal mix institution & private	2
Private only	1

Table 6: Time devoted to teaching

**Hours per week**	**Number of participants**
1-5	1
6-10	1
11-15	2
16-20	1
21-25	3
Intermittent (once every few months; can be intensive)	2

Table 7: Income

**Type of income**	**Number of participants**
Hourly fees	7
Hourly fees and salary	1
No answer	2

Table 8: Students taught

**Type of students***	**Number of participants**
Adolescents	2
University /conservatory	8
Young professionals & adults	2
Professionals	1

*More than one category could be chosen

Table 9: Personal on-going training

**Type of training**	**Number of participants**
Voice lessons	5
Lessons and coaching	2
With conductors	1
None in last few years	2

Table 10: Summary of participants' background

Participant	Professional category	Years of experience		Training	Teaching	Teaching hours	Income	Students	On-going training
		Singing	Teaching						
Anna	'A' opera	33	5	Postgraduate	Mostly private	Intermittent; can be intensive	Hourly fees	Young professionals &adults	Voice lessons
Bartolo	'A' opera	35	8	Postgraduate	Mostly institution	Intermittent; can be intensive	Hourly fees	University/ conservatory	Voice lessons
Cherubino	Music theatre	18	3	Private training	Mostly institution	6	Hourly fees	University/ conservatory	None in last few years
Count	Institution	25	32	Postgraduate	Mostly institution	22-25	No info	University/ conservatory	Lessons & coaching
Countess	Studio	8	8	Postgraduate	Mostly private	16	Hourly fees	Adolescents, university/ conservatory	Voice lessons
Fiordiligi	Institution	27	27	Postgraduate	Mostly institution	24-25	Hourly fees & salary	University/ conservatory	None in last few years
Giovanni	'A' opera	34	11	Postgraduate	Almost equal mix	15	Hourly fees	University/ conservatory	Lessons & coaching
Marcellina	National	36	5	Postgraduate	Mostly institution	5.5	Hourly fees	University/ conservatory	With conductors
Susanna	National	22	8	Postgraduate	Almost equal mix	12-15	No info	Adolescents, university/ conservatory, young professionals & adults	Voice lessons
Zerlina	Studio	20	30	Postgraduate	Private only	24	Hourly fees	Professionals	Voice lessons

*In order to help preserve the anonymity of participants, their country of origin is omitted in this table.
